# Supplementary material for: Use of Danish National Somatic Cell Count Data to Assess the Need for Dry-Off Treatment in Holstein Dairy Cattle
Source: Animals (Basel). 2023 Aug 4;13(15):2523. doi: 10.3390/ani13152523 (PMC10416964; doi:10.3390/ani13152523)
Supplement: Supplementary file 1 [file animals-13-02523-s001.zip › animals-2496339-Supplementary Material.pdf]

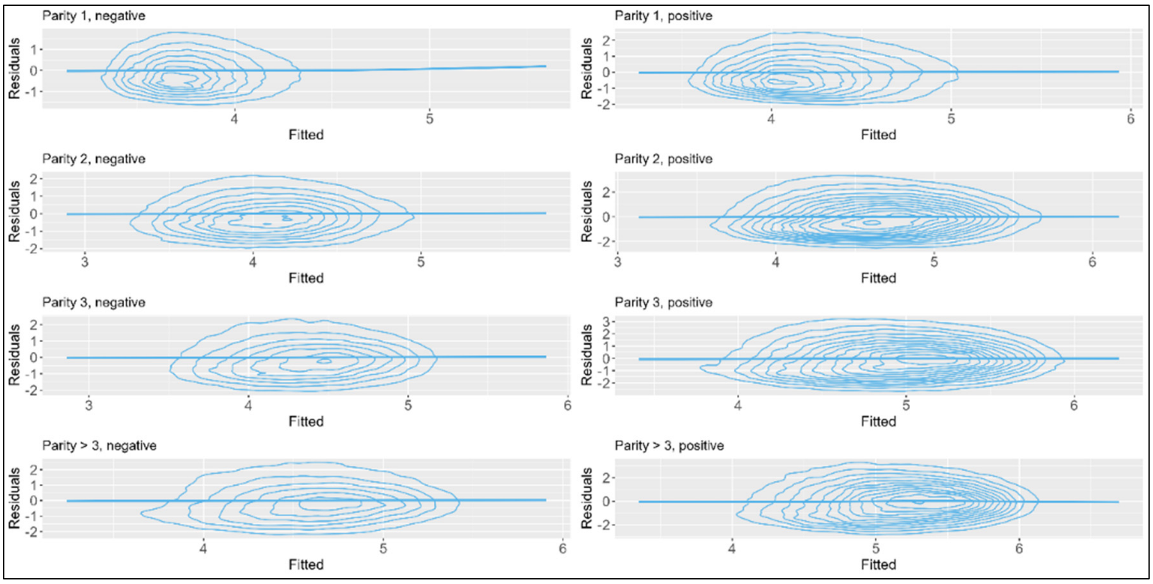

Figure S1. Density plot presenting the residuals vs fitted for the eight different models: to the left, PCR negative for Parities 1, 2, 3, and >3, and to the right, PCR positive for Parities 1, 2, 3, and >3.
